# Endogenous Hydrogen Sulfide Persulfidates Caspase-3 at Cysteine 163 to Inhibit Doxorubicin-Induced Cardiomyocyte Apoptosis

**DOI:** 10.1155/2022/6153772

**Published:** 2022-05-04

**Authors:** Xiaoyun Ye, Yingying Li, Boyang Lv, Bingquan Qiu, Shangyue Zhang, Hanlin Peng, Wei Kong, Chaoshu Tang, Yaqian Huang, Junbao Du, Hongfang Jin

**Affiliations:** ^1^Department of Pediatrics, Peking University First Hospital, Beijing 100034, China; ^2^Department of Cardiovascular Medicine, Children's Hospital Affiliated to Zhengzhou University/Children's Hospital of Henan Province, Zhengzhou, China; ^3^Department of Physiology and Pathophysiology, Peking University Health Science Center, Beijing 100191, China; ^4^Key Laboratory of Molecular Cardiovascular Sciences, Ministry of Education, Beijing 100191, China

## Abstract

Doxorubicin (DOX) is an efficient antitumor anthracycline drug, but its cardiotoxicity adversely affects the prognosis of the patients. In this study, we explored whether endogenous gasotransmitter hydrogen sulfide (H_2_S) could protect against DOX-induced cardiomyocyte apoptosis and its mechanisms. The results indicated that DOX significantly downregulated endogenous H_2_S production and endogenous synthetase cystathionine *γ*-lyase (CSE) expression and obviously stimulated the apoptosis in H9C2 cells. The supplement of H_2_S donor sodium hydrosulfide (NaHS) or overexpression of CSE inhibited DOX-induced H9C2 cell apoptosis. DOX enhanced the activities of caspase family members in cardiomyocytes, while NaHS attenuated DOX-enhanced caspase-3, caspase-2, and caspase-9 activities by 223.1%, 73.94%, and 52.29%, respectively. Therefore, taking caspase-3 as a main target, we demonstrated that NaHS or CSE overexpression alleviated the cleavage of caspase-3, suppressed caspase-3 activity, and inhibited the cleavage of poly ADP-ribose polymerase (PARP). Mechanistically, we found that H_2_S persulfidated caspase-3 in H9C2 cells and human recombinant caspase-3 protein, while the thiol-reducing agent dithiothreitol (DTT) abolished H_2_S-induced persulfidation of caspase-3 and thereby prevented the antiapoptotic effect of H_2_S on caspase-3 in H9C2 cells. The mutation of caspase-3 C148S and C170S failed to block caspase-3 persulfidation by H_2_S in H9C2 cells. However, caspase-3 C163S mutation successfully abolished the effect of H_2_S on caspase-3 persulfidation and the corresponding protection of H9C2 cells. Collectively, these findings indicate that endogenous H_2_S persulfidates caspase-3 at cysteine 163, inhibiting its activity and cardiomyocyte apoptosis. Sufficient endogenous H_2_S might be necessary for the protection against myocardial cell apoptosis induced by DOX. The results of the study might open new avenues with respect to the therapy of DOX-stimulated cardiomyopathy.

## 1. Introduction

Doxorubicin (DOX) is commonly used for the chemotherapy of a variety of solid tumors and hematological tumors. However, its cardiotoxicity is an important issue in the field of oncocardiology. With the increase in the cumulative dose of DOX, the prevalence of congestive heart failure also increases [1]. Moreover, the mortality rate of patients treated with DOX-induced heart failure was relatively increased [2]. Since cardiomyocyte apoptosis is an important pathological basis of DOX-induced cardiomyopathy [3], it is of great significance to clarify the underlying mechanism for improving cardiac function and life quality in patients treated with DOX. Previous data have shown that the inhibition of DNA/RNA/protein synthesis in cardiomyocytes [4, 5], oxidative stress injury [6], lipid peroxidation [7], calcium homeostasis disorders, endoplasmic reticulum stress [8], mitochondrial dysfunction [9], increased inflammatory mediators [10], and abnormal autophagy regulation [11] are all important mechanisms for DOX-induced apoptosis in myocardial cells. However, the molecular mechanisms underlying apoptosis in cardiomyocytes exposed to DOX have not yet been fully elucidated and urgently require further in-depth exploration.

Cysteine aspartic acid-specific proteases (caspases) exist in the cytoplasm, and their active sites all contain cysteines which can specifically cleave peptide bonds on target aspartic acid residues and play a significant role in apoptosis. A previous study has shown that DOX upregulates the mRNA levels of caspase-6 in rat cardiomyocytes [12]. Caspase-6, an effector caspase, depends on caspase-3 activation [13, 14] and cleaves the lamina required for cell morphology, accelerating chromatin condensation and apoptotic body formation [14]. Caspase-6 can also activate caspase-8 [13]. In the cytoplasm, activated caspase-8 cleaves BH3-interacting domain death agonist (Bid) to form the active molecule truncated Bid (tBid), which facilitates cytochrome c release from mitochondria [15]. DOX-stimulated oxidative stress decreases the Bcl-2/Bax ratio [12], resulting in changes in the structure of the mitochondrial membrane to facilitate cytochrome c translocation to the cytosol. Cytochrome c triggers the cleavage of caspase-9, which further increases the activity of the effector molecule caspase-3 [16, 17]. DOX can also upregulate the expression of death receptors, such as TNF receptor 1 [18], Fas [19], DR4, and DR5 [20], which combine with the corresponding free ligand to form a specific complex that initiates caspase-8 to directly activate caspase-3 [21]. Caspase-4 and caspase-12 are highly homologous, and both exist in the endoplasmic reticulum as precursors [22]. DOX-stimulated endoplasmic reticulum stress activates caspase-4 and caspase-12 and promotes the release of the two caspases from the endoplasmic reticulum to the cytoplasm. And then, the cytoplasmic activated caspase-4 and caspase-12 initiate the caspase cascade including caspase-9 and caspase-3, ultimately leading to apoptosis [22]. The above studies suggest that caspases are key molecules in the mechanisms of DOX-stimulated cardiomyocyte apoptosis. Previous studies have shown that the phosphorylation of caspase-9 at Thr-125 and Ser-196 inhibits the activation of caspase-9 [23, 24], while phosphorylation at Tyr-251 enhances the activity of caspase-9 and accelerates apoptosis [25]. The glutathionylation of caspase-3 at Cys-220 and Cys-163 suppresses its activity [26]. These findings suggest that the posttranslational modification of caspases is important for the regulation of their activities.

Hydrogen sulfide (H_2_S) is a newly identified gasotransmitter [27–30]. It is primarily generated endogenously by cystathionine *γ*-lyase (CSE) catalysis in the cardiovascular system. H_2_S plays a major regulatory role in maintaining cardiovascular function and structure [31, 32]. Previous studies have shown that persulfidation targeting sulfhydryl groups at key cysteine residues of proteins is an important molecular mechanism by which H_2_S regulates protein function [33, 34]. H_2_S persulfidates CSE to enhance its activity and decrease homocysteine levels to reduce atherosclerosis [35]. H_2_S inhibits IKK*β* activity through persulfidating Cys-179 on IKK*β*, inhibiting the inflammatory response of pulmonary artery endothelial cells [33]. Previous research by our group demonstrated that H_2_S donors significantly improved cardiac function and attenuated cardiac pathological micro- and ultrastructural changes in DOX-treated rats, including inflammatory cell infiltration and mitochondrial edema [36]. However, it is not clear whether and how H_2_S regulates caspase activity to inhibit the apoptosis of DOX-induced cardiomyocytes.

Therefore, in the present study, we examined the impact of endogenous H_2_S on caspases and the underlying molecular mechanism to reveal new insights into the development of DOX-stimulated cardiomyocyte apoptosis.

## 2. Materials and Methods

### 2.1. Cell Culture and Experimental Treatments

The rat embryonic cardiomyocyte H9C2 cell line was procured from Wuhan Procell Company (Wuhan, China). Cells were cultured in DMEM supplemented with 10% FBS (Thermo, Gibco, United States) and 1% penicillin/streptomycin (37°C, 5% CO_2_). When H9C2 cells reached semiconfluence, they were starved in the basal DMEM for 8 h. The H9C2 cell model was prepared by exposure to DOX (0.3 *μ*mol/L) for 24 h [37], and H_2_S donor sodium hydrosulfide (NaHS, 100 *μ*mol/L) or CSE overexpression was used to provide sufficient H_2_S levels.

### 2.2. Cell Viability Measured Using the Cell Counting Kit-8 (CCK-8) Assay

Cell viability was analyzed using an enhanced CCK-8 kit (Beyotime, Shanghai, China) [38]. Cells were plated at a density of 5 × 10^3^/well in microplates. H9C2 cells were exposed to DOX (0, 0.1, 0.2, 0.3, 0.4, and 0.5 *μ*mol/L) after starvation in basal DMEM for 8 h. Twenty-four hours after treatment, the basic medium was changed to medium containing 10% CCK-8 and cultured for 2 h at 37°C in the dark. Cell-free wells with reagent were used as blank controls, and absorbance values were measured at 450 nm. Cell viability was calculated using the following equation: (OD stimulated − OD blank)/(OD unstimulated − OD blank) × 100%.

### 2.3. Apoptosis Assessment Using a TdT-Mediated dUTP Nick-End Labeling (TUNEL) Kit

A TUNEL kit (Roche, USA) was applied to assess the apoptosis in H9C2 cells [39]. The cells were gently rinsed with PBS after discarding the supernatant and fixed in 4% paraformaldehyde for 15 min. Afterward, they were permeabilized for 30 min following incubation with the TUNEL reagent for 1 h at 37°C protected from light. Finally, nuclei were labeled using DAPI dye. Fluorescence images were captured using a confocal laser scanning microscope (Olympus, Japan). Green fluorescence represents the TUNEL-labeled signal marking apoptotic cells. The apoptotic index was described as the percentage of the number of TUNEL-labeled nuclei (green) to the total DAPI-labeled nuclei (blue).

### 2.4. Caspase Activity Detected Using the Caspase Family Colorimetric Assay Kit in H9C2 Cells

The activities of caspases were assessed using a series of caspase activation assay kits (Applygen, Beijing, China) according to the manufacturer's instructions [38]. Caspase activity kits in the present study included caspase-1 colorimetric assay kit (C1111), caspase-2 colorimetric assay kit (C1112), caspase-3 colorimetric assay kit (C1113), caspase-4 colorimetric assay kit (C1114), caspase-5 colorimetric assay kit (C1115), caspase-6 colorimetric assay kit (C1116), caspase-8 colorimetric assay kit (C1118), and caspase-9 colorimetric assay kit (C1119). The active caspases recognize specific peptides and cleave the substrate peptides at specific aspartic acid residues. Different caspase activity kits contain synthetic corresponding peptides in which the aspartic acid residue is conjugated with p-nitroanilide (pNA). The active caspase cleaves the peptide-pNA compound at the aspartic acid residue and releases free pNA which has a maximum absorbance at 405 nm. One unit of caspase enzymatic activity is defined as the hydrolysis of substrate peptide-pNA at 37°C for 1 h to release 1 nmol of free pNA. In brief, the cells were rinsed gently with precooled PBS and incubated in lysis solution on ice at 4°C for 15 min. The total protein was harvested into centrifuge tubes and chilled on ice for 10 min, followed by a centrifugation at 12000 *g* and 4°C. The sample and the substrate were added to a 96-well plate in sequence and then incubated for 2 h at 37°C protected from light. The pNA content was detected by measuring the absorbance OD value at 405 nm. Caspase activity was calculated according to the pNA standard curve and the OD value of the sample. The protein concentration was detected by the Bradford method and used to normalize the caspase activity.

### 2.5. H_2_S Detection Using a Fluorescent Probe in H9C2 Cells

The H_2_S-specific fluorescent probe SF7-AM (Cayman, Ann Arbor, USA) was used to determine the H_2_S content in cells [40]. Cells were gently rinsed with PBS after discarding the medium. Subsequently, the cells were incubated with 2.5 *μ*mol/L H_2_S fluorescent probe in the incubator for 30 min protected from light. The cells were incubated in 4% paraformaldehyde for 20 min at 25°C. Green fluorescence was captured using a confocal laser scanning microscope, indicating H_2_S content in the cells. DAPI dye was used for nuclear staining.

### 2.6. CSE Overexpression in H9C2 Cells

An hCSE lentivirus (Cyagen, Guangzhou, China) was constructed to induce CSE overexpression. Empty vector lentivirus (EV) was used as a control. Briefly, 3 × 10^5^ cells were plated into a T25 flask and allowed to grow to 50% confluence, where fresh medium was added, and the cells were infected with the virus (MOI = 10). Cells were replenished with fresh complete medium 24 h after transfection and cultured for another 48 h. Stable CSE-overexpressing H9C2 cells were selected by treatment with G418 (100 *μ*g/mL) for 2 w.

### 2.7. Caspase-3 Knockdown in H9C2 Cells

Caspase-3 knockdown H9C2 cells were obtained by transfecting lentivirus containing caspase-3 shRNA and green fluorescent protein (GFP) cDNA (Cyagen, Guangzhou, China). As a negative control, lentiviruses containing scrambled shRNA were used. A total of 3 × 10^5^ cells were seeded into a T25 flask and grown to 50% confluence, replaced with fresh medium, and then infected with the virus (MOI = 10). Caspase-3 shRNA lentivirus was added in equal amounts 24 h after transfection, and cells were given fresh complete medium 48 h after transfection. Using fluorescence microscopy, GFP green fluorescence was observed 96 h after transfection. Stable caspase-3 shRNA-transfected H9C2 cells were selected by treatment with puromycin (1 *μ*g/mL) for 2 w.

### 2.8. Plasmid Transfection

Caspase-3 wild-type (WT) plasmid and mutant plasmids C148S (Cys-148 mutated to serine), C163S (Cys-163 mutated to serine), and C170S (Cys-170 mutated to serine) with pcDNA3.1+ as the vector were constructed (Sangon Biotech, Shanghai, China). Caspase-3 knockdown H9C2 cells were plated in cell culture plates and then transfected using a Lipofectamine 3000 reagent (Thermo, Carlsbad, USA) when cell confluence reached 50%. The DNA and transfection agents were diluted in Opti-MEM (Gibco, USA) according to the instructions, mixed, and incubated for 15 min. The medium was replaced with fresh complete medium, and the DNA-lipid complex was added to the cells. The subsequent experiments were conducted 48 h after transfection.

### 2.9. Caspase-3 Persulfidation Detection Using Biotin Switch Analysis (BSA)

The BSA method was used to determine the persulfidation of caspase-3 in H9C2 cells [34]. DOX-treated H9C2 cells were divided into DOX, DOX+NaHS, and DOX+NaHS+dithiothreitol (DTT) groups. DOX-untreated H9C2 cells were divided into control, NaHS, and NaHS+DTT groups. For the H9C2 cells, the treatment conditions included the incubation with 100 *μ*mol/L NaHS for 2 h, 200 *μ*mol/L DTT for 15 min, and 0.3 *μ*mol/L DOX for 24 h. After the experiment, the cells were gently rinsed with PBS, and then, 120 *μ*L of the cell lysis was added and incubated for 20 min at 4°C. The cells were collected into an EP tube and then centrifuged at 13000 *g* at 4°C to obtain the supernatant. Ten microliters of the supernatant were mixed with 2x SDS loading buffer and boiled to detect the total caspase-3 using denaturing SDS-PAGE electrophoresis. The remaining supernatant was used to analyze the caspase-3 persulfidation. First, the supernatant was incubated with 2.5% SDS and 20 mM S-methyl methanethiosulfonate (MMTP) for 20 min at 50°C. And then precooled acetone was added and kept at -20°C for 2 h to precipitate the protein. The precipitated protein pellets were collected after the centrifugation for 10 min at 4°C. Subsequently, the precipitants were dissolved in 200 *μ*L of the cell lysis buffer containing 3 *μ*L of EZ-linked iodoacetyl-PEG2 biotin (10 mg/mL) and then incubated on a shaker at 4°C for 12 h. To enrich the persulfidated protein, 6 *μ*L of UltraLink Immobilized NeutrAvidin was used to combine the biotinylated proteins and incubated for 4 h at 4°C. The precipitation was collected by centrifuging at 5000 *g* at 4°C for 10 min. After rinsing 6 times with PBS containing 0.001% SDS, the samples were mixed with 1x *β*-mercaptoethanol-free SDS loading buffer, boiled, centrifuged at 5000 *g* to collect the persulfidated proteins, and loaded onto 10% SDS–PAGE to analyze caspase-3 persulfidation.

Recombinant human caspase-3 protein (Origene, Rockville, USA) was divided into control, NaHS, and NaHS+DTT groups. In the NaHS group, 0.15 *μ*g of caspase-3 protein was incubated with 100 *μ*mol/L NaHS for 30 min at 37°C, while in the NaHS+DTT group, the protein was treated with NaHS and 1 *μ*mol/L DTT for the last 15 min. The protocol for analyzing total and persulfidated caspase-3 protein was the same as that in the cell experiments.

### 2.10. Protein Expression Detected by Western Blot Analysis

The expression of proteins in H9C2 cells was analyzed by western blotting analysis as described in a previous study [41]. Briefly, H9C2 cells were collected and rinsed gently with precooled PBS. Each well of the 6-well plate was lysed using 60 *μ*L of cell lysis buffer on ice for 30 min. The supernatant was obtained after the centrifugation of 12000 *g* at 4°C, and 1 *μ*L of the supernatant was used for the quantification of the protein concentration using the BCA method. The remaining protein was denatured in 2x loading buffer for 10 min. The denatured proteins (15-30 *μ*g) were separated using 10%-15% SDS-PAGE electrophoresis and transferred onto nitrocellulose (NC) membranes. Nonspecific binding was blocked using 5% skimmed milk at 25°C for 1 h. The NC bands were incubated at 4°C for 12 h with the primary antibodies diluted in PBST: caspase-3 (1 : 1000, AC030, Beyotime, China), cleaved caspase-3 (1 : 500, AC030, Beyotime), PARP (1 : 1000, AP102, Beyotime), CSE (1 : 1000, 12217-1-AP, Proteintech, China), *β*-tubulin (1 : 4000, TA-10, ZSGB, China), and *β*-actin (1 : 4000, TA-09, ZSGB, China). After washing with PBST, the bands were immersed with the corresponding horseradish peroxidase-labeled secondary antibodies at a working concentration of 1 : 5000 at 25°C for 1 h. Finally, the NC bands were incubated with ECL chemiluminescence reagents. A FluorChem M chemiluminescence imaging analysis system was used to take pictures, and ImageJ software (version 1.4.3) was used for gray value analysis.

### 2.11. Statistical Analysis

SPSS 17.0 (IBM, USA) and GraphPad (GraphPad, CA, USA) were applied for the statistical analysis. All data are expressed as the mean ± SEMs. A comparison among multiple groups was performed using ANOVA followed by Tukey's test if the data were normally distributed or followed by a Dunnett T3 procedure if the data were not normally distributed. *p* < 0.05 was regarded as statistically significant.

## 3. Results

### 3.1. H_2_S Donor or CSE Overexpression Prevented DOX-Stimulated Apoptosis in Cardiomyocytes

**Figure 1:**
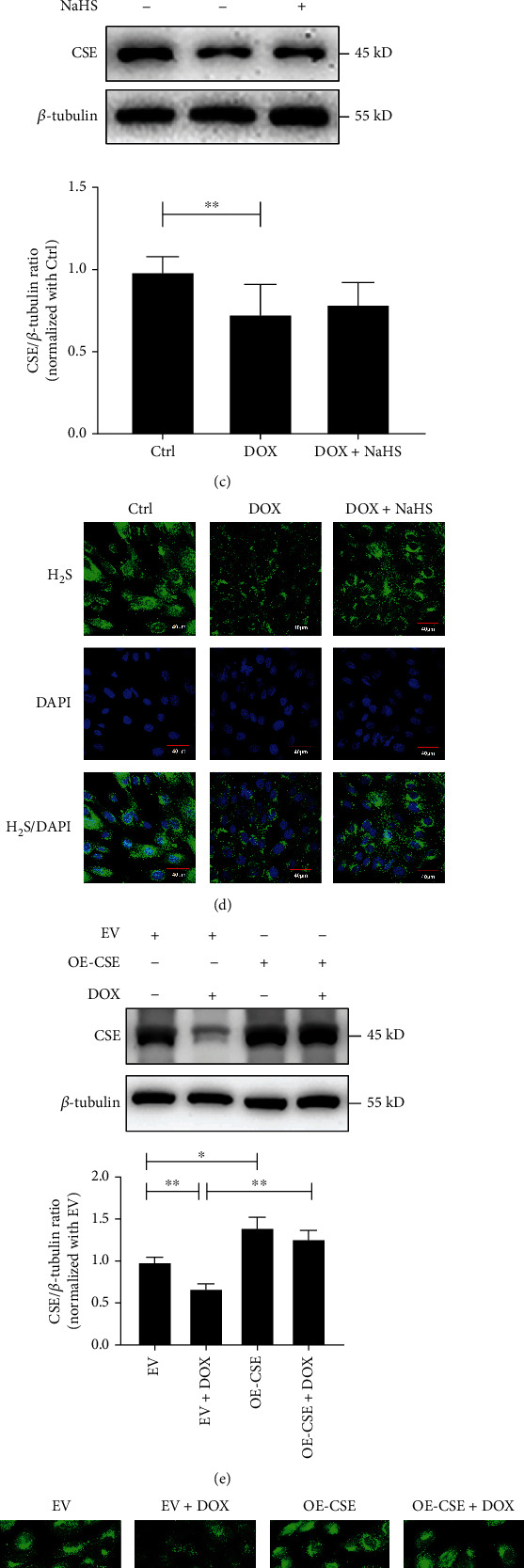
DOX induced H9C2 cell apoptosis by downregulating the endogenous CSE/H_2_S pathway. (a) CCK-8 assay was used to detect the viability of H9C2 cells treated with different concentrations of DOX (0-0.5 *μ*mol/L) for 24 h (*n* = 15). (b) The TUNEL assay was used to detect the DOX-induced apoptosis in H9C2 cells. The blue fluorescence indicated the nuclei. The green fluorescence indicated the TUNEL-positive nuclei. The superposition of blue fluorescence and green fluorescence indicated apoptotic cells (magnification, ×600; scale bar = 40 *μ*m, *n* = 15). The representative images were from three independent experiments. For each independent experiment, at least 5 fields were observed and counted under a confocal laser scanning microscope in each group. (c) Western blot analysis was used to detect the expression of CSE protein in DOX-treated H9C2 cells (*n* = 9). (d) Fluorescence probe assay was used to detect H_2_S content in DOX-treated H9C2 cells, where green fluorescence indicated H_2_S and blue fluorescence indicated nuclei (magnification, ×600; scale bar = 40 *μ*m, *n* = 15). (e) Western blot analysis was used to detect the expression of CSE protein in CSE-overexpressed H9C2 cells with or without DOX treatment (*n* = 9). (f) H_2_S content in CSE-overexpressed H9C2 cells with or without DOX treatment by fluorescence probe assay, where green fluorescence indicated H_2_S and blue fluorescence indicated cell nuclei (magnification, ×600; scale bar = 40 *μ*m, *n* = 15). (g) TUNEL assay was used to detect the apoptosis of CSE-overexpressed H9C2 cells with or without DOX treatment, where blue fluorescence indicated the nuclei, green fluorescence indicated the TUNEL-positive nuclei, and the superposition of blue fluorescence and green fluorescence indicated apoptotic cells (magnification, ×600; scale bar = 40 *μ*m, *n* = 15). The representative images were from three independent experiments. For each independent experiment, at least 5 fields were observed and counted under a confocal laser scanning microscope in each group. The data were shown as means ± SEMs, ^∗^*p* < 0.05, ^∗∗^*p* < 0.01. Ctrl: control; DOX: doxorubicin; DOX+NaHS: doxorubicin+sodium hydrosulfide; EV: empty vector; EV+DOX: empty vector+doxorubicin; OE-CSE: CSE overexpression; OE-CSE+DOX: CSE overexpression+doxorubicin.

Cells were treated with DOX (0.1-0.5 *μ*mol/L) for 24 h, and the viability of H9C2 cells was determined using the CCK-8 assay. The data indicated that cell viability treated with 0.1, 0.2, 0.3, 0.4, and 0.5 *μ*mol/L DOX was decreased by 22.77%, 33.24%, 47.79%, 49.8%, and 52.28%, respectively, compared to the controls (*p* < 0.01, Figure 1(a)). By using 0.3 *μ*mol/L DOX as an intervention dose in the following experiment, we showed that compared to the controls, the proportion of TUNEL-labeled cells treated with DOX was increased by 85.94%, while CSE protein level decreased by 26.18% (*p* < 0.01, Figures 1(b) and 1(c)). A fluorescence probe assay was used to quantify H_2_S content in cells. A marked decrease in H_2_S content in DOX-stimulated H9C2 cells was observed, as evidenced by a significant decrease in H_2_S-specific green fluorescence compared to the controls (Figure 1(d)). These findings suggest that DOX downregulates the H_2_S/CSE pathway in cells and induces H9C2 cell apoptosis.

To explore the impact of endogenous H_2_S reduction on apoptosis in DOX-stimulated cardiomyocytes, treatment with the H_2_S donor or overexpression of the H_2_S-producing enzyme CSE was performed to increase the H_2_S content in DOX-treated H9C2 cells. The results of H_2_S donor NaHS (100 *μ*mol/L) treatment revealed that the H_2_S fluorescence intensity of the cells was increased, while the proportion of the TUNEL-labeled cells decreased by 65.97% (*p* < 0.01, Figures 1(b) and 1(d)) in the DOX+NaHS group compared to the DOX group. The expression of CSE and H_2_S content in CSE-overexpressing cells was significantly increased (Figures 1(e) and 1(f)) compared to that in the EV group. In the cells transfected with EV, the treatment of DOX decreased the expression of CSE and H_2_S content (Figures 1(e) and 1(f)), but increased the proportion of apoptotic cells (*p* < 0.01, Figure 1(g)). However, DOX treatment did not affect CSE protein expression, H_2_S content, or the proportion of TUNEL-positive cells (Figures 1(e)–1(g)) in CSE-overexpressed cells. These results suggest that sufficient H_2_S content in H9C2 cells prevents the cardiomyocyte from DOX-stimulated apoptosis.

### 3.2. H_2_S Inhibited DOX-Stimulated Caspase-3 Activation in Cardiomyocytes

**Figure 2:**
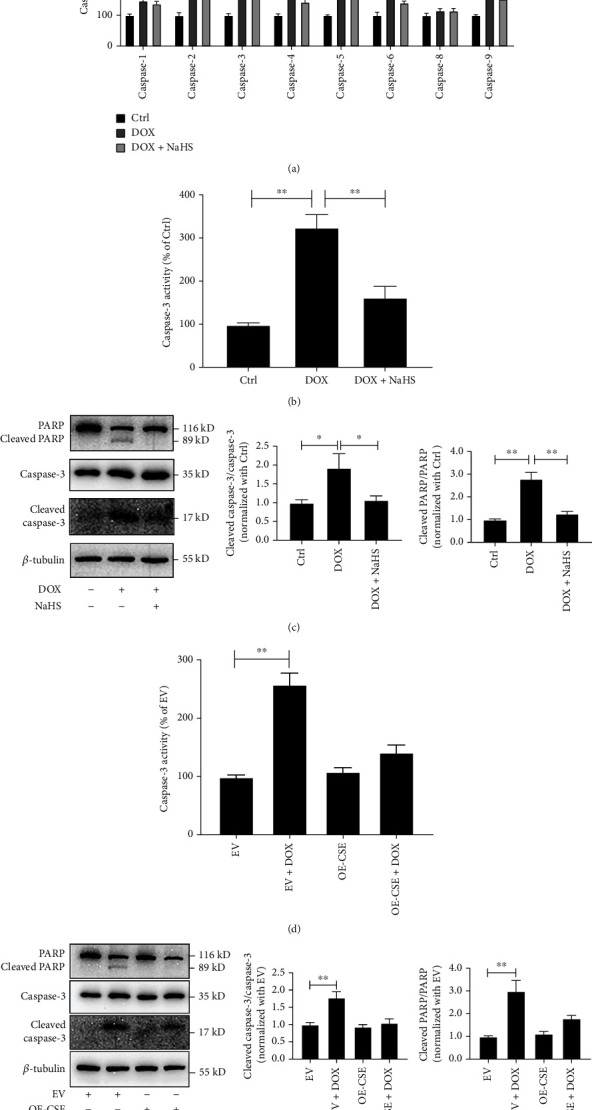
H_2_S inhibited caspase-3 activity in DOX-treated cardiomyocytes. (a) Colorimetric assay was used to detect the caspase activity in H9C2 cells. (b) Colorimetric assay was used to detect the caspase-3 activity of H9C2 cells treated with NaHS and DOX. (c) Western blot analysis was used to detect the ratio of cleaved caspase-3/caspase-3 and cleaved PARP/PARP in H9C2 cells treated with NaHS and DOX. (d) Colorimetric assay was used to detect the caspase-3 activity in CSE-overexpressed H9C2 cells with DOX treatment. (e) Western blot analysis was used to detect the ratio of cleaved caspase-3/caspase-3 and cleaved PARP/PARP in CSE-overexpressed H9C2 cells with DOX treatment. Data were shown as mean ± SEMs, ^∗^*p* < 0.05, ^∗∗^*p* < 0.01, *n* = 9. Ctrl: control; DOX: doxorubicin; DOX+NaHS: doxorubicin+sodium hydrosulfide; EV: empty vector; EV+DOX: empty vector+doxorubicin; OE-CSE: CSE overexpression; OE-CSE+DOX: CSE overexpression+DOX.

The screening of caspase enzymatic activity revealed that the activities of caspase-1, caspase-2, caspase-3, caspase-4, caspase-5, caspase-6, and caspase-9 in DOX-treated cells were significantly increased by 47.97%, 129.30%, 335.00%, 88.51%, 111.5%, 61.27%, and 106.5%, respectively, compared to those in the controls (*p* < 0.01, Figure 2(a)), suggesting that DOX extensively activated the members of the caspase family in association with cardiomyocyte apoptosis. Furthermore, caspase-3, caspase-2, and caspase-9 activities in cells of the DOX+NaHS group were decreased by 223.1% (*p* < 0.01), 73.94% (*p* < 0.05), and 52.29% (*p* < 0.01) compared with those of the DOX group, respectively (Figure 2(a)). These data suggested that caspase-3, caspase-2, and caspase-9 were the possible target molecules of H_2_S against cardiomyocyte apoptosis, and caspase-3 was most significantly regulated by H_2_S.

Next, we examined the regulatory effect of H_2_S on caspase-3 in DOX-stimulated H9C2 cells. The data indicated that the cleaved caspase-3/caspase-3 ratio was increased by 94.11% (*p* < 0.05), caspase-3 activity increased by 227.8% (*p* < 0.01), and the caspase-3 downstream cleaved PARP/PARP ratio increased by 182.7% (*p* < 0.01) in the cells of the DOX group compared with the controls (Figures 2(b) and 2(c)). Compared with the DOX group, the cleaved caspase-3/caspase-3 ratio was decreased by 86.17% (*p* < 0.05), caspase-3 activity decreased by 163.8% (*p* < 0.01), and the cleaved PARP/PARP ratio decreased by 154.4% (*p* < 0.01) in the cells of the DOX+NaHS group (Figures 2(b) and 2(c)). Similarly, compared with the EV group, the cleaved caspase-3/caspase-3 ratio was increased by 178.7% (*p* < 0.01), caspase-3 activity increased by 160.3% (*p* < 0.01), and the cleaved PARP/PARP ratio increased by 201.4% (*p* < 0.01) in the EV+DOX group. No significant differences were detected in the above indicators between the OE-CSE and OE-CSE+DOX groups (Figures 2(d) and 2(e)). These results suggested that sufficient H_2_S ameliorated DOX-induced caspase-3 activity in H9C2 cells.

### 3.3. H_2_S Inhibited Caspase-3 Activity and Cardiomyocyte Apoptosis by Persulfidating Caspase-3

**Figure 3:**
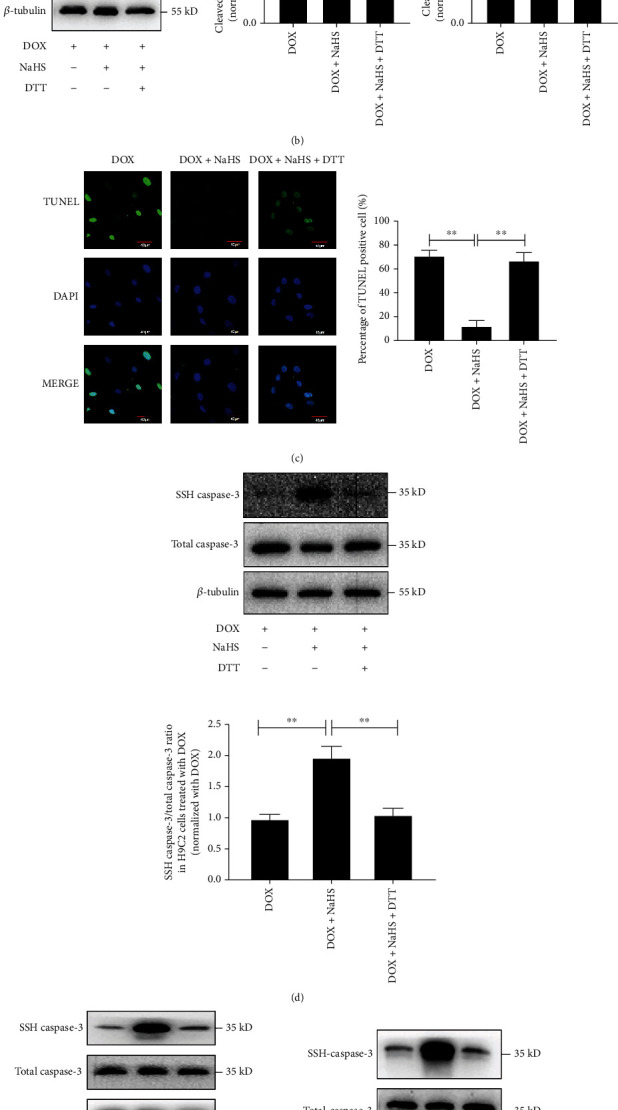
H_2_S inhibited caspase-3 activity and reduced H9C2 cell apoptosis by persulfidating caspase-3. (a) Colorimetric assay was used to detect caspase-3 activity in H9C2 cells (*n* = 9). (b) Western blot analysis was used to detect the ratio of cleaved caspase-3/caspase-3 and cleaved PARP/PARP in H9C2 cells (*n* = 12). (c) The TUNEL assay was used to detect the apoptosis of H9C2 cells, where blue fluorescence indicated the nuclei, the green fluorescence indicated the TUNEL-positive nuclei, and the superposition of blue fluorescence and green fluorescence indicated the apoptotic cells (magnification, ×600; scale bar = 40 *μ*m, *n* = 15). The representative images were from three independent experiments. For each independent experiment, at least 5 fields were observed and counted under a confocal laser scanning microscope in each group. (d) Biotin switch analysis was used to detect the persulfidation of caspase-3 protein in DOX-treated H9C2 cells and *β*-actin as an internal control (*n* = 9). (e) Biotin switch analysis was used to detect the persulfidation of caspase-3 protein in DOX-untreated H9C2 cells and *β*-actin as an internal control (*n* = 9). (f) Biotin switch analysis was used to detect the persulfidation of human recombinant caspase-3 protein (*n* = 9). The data were shown as mean ± SEMs, ^∗∗^*p* < 0.01. DOX: doxorubicin; DOX+NaHS: doxorubicin+sodium hydrosulfide; DOX+NaHS+DTT: doxorubicin +sodium hydrosulfide+dithiothreitol; Ctrl: control; NaHS: sodium hydrosulfide; NaHS+DTT: sodium hydrosulfide+dithiothreitol.

To explore the molecular mechanism by which H_2_S inhibited caspase-3 activity and cardiomyocyte apoptosis, H9C2 cells were divided into DOX, DOX+NaHS, and DOX+NaHS+DTT groups. Compared with the DOX+NaHS group, caspase-3 activity was enhanced by 77.05%, the ratio of cleaved caspase-3/caspase-3 increased by 48.74%, the ratio of cleaved PARP/PARP increased by 45.61%, and the proportion of TUNEL-positive cells increased by 55.22% (*p* all <0.01) (Figures 3(a)–3(c)) in the cells of the DOX+NaHS+DTT group. These findings suggested that the sulfhydryl reducing agent DTT abolished the suppressive effect of H_2_S on DOX-stimulated caspase-3 activity and cardiomyocyte apoptosis in H9C2 cells. Therefore, H_2_S might inhibit caspase-3 activity by targeting the sulfhydryl group.

To demonstrate how H_2_S affected the sulfhydryl group of caspase-3, BSA method was used to detect the persulfidation of caspase-3 in the DOX-treated H9C2 cells.  Figure 3(d) shows that the persulfidation of caspase-3 in the cells of the DOX+NaHS group was increased by 99.7% compared with the DOX group (*p* < 0.01), while the H_2_S-induced caspase-3 persulfidation was weakened in the cells of the DOX+NaHS+DTT group, accompanied by the reactivated caspase-3 (*p* < 0.01). Furthermore, the treatment of NaHS also induced a significant persulfidation of caspase-3 in the DOX-untreated H9C2 cells and purified human caspase-3 protein, which was abolished by the intervention with DTT (*p* all <0.01) (Figures 3(e) and 3(f)). The abovementioned results suggested that H_2_S might suppress caspase-3 activity by persulfidating the sulfhydryl group of caspase-3 protein to inhibit the cardiomyocyte apoptosis.

### 3.4. Cys-163 Was the Key Site for H_2_S to Persulfidate Caspase-3

**Figure 4:**
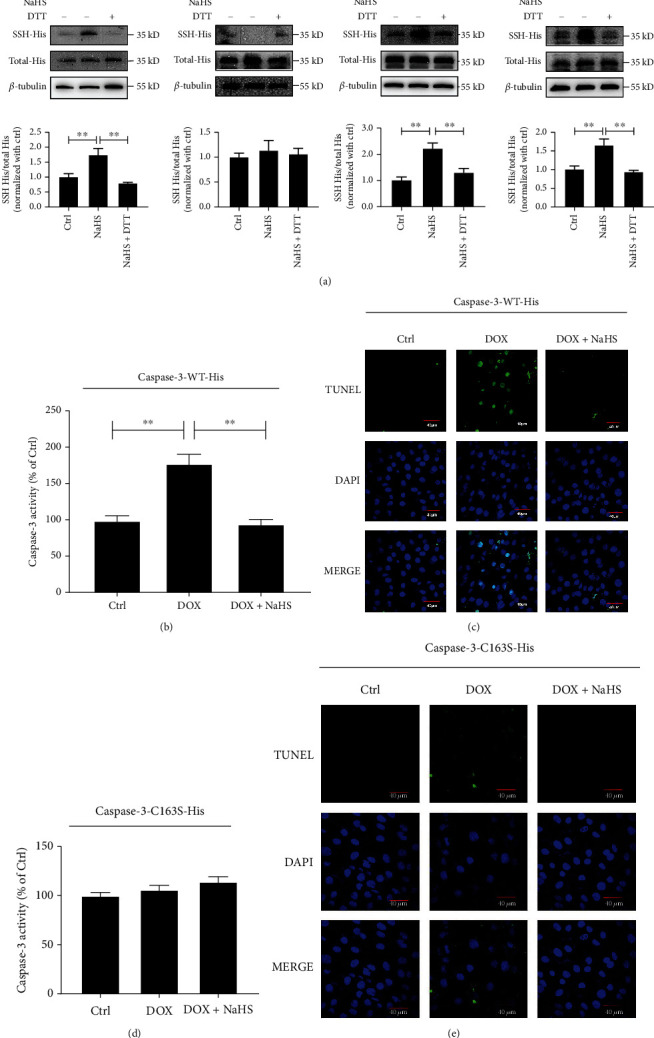
H_2_S persulfidated caspase-3 at Cys-163 to inhibit its activity and then reduced DOX-induced cardiomyocyte apoptosis. (a) Biotin switch analysis was used to detect the persulfidation of caspase-3 in H9C2 cells transfected with caspase-3-WT-His plasmid (*n* = 9), caspase-3-C163S-His (*n* = 12) plasmid, caspase-3-C148S-His plasmid (*n* = 12), or caspase-3-C170S-His (*n* = 9) plasmid. (b) Colorimetric assay was used to detect the caspase-3 activity in H9C2 cells transfected with caspase-3-WT-His plasmid (*n* = 9). (c) The TUNEL assay was used to detect the apoptosis of H9C2 cells transfected with caspase-3-WT-His plasmid. The blue fluorescence indicated the nuclei, the green fluorescence indicated the TUNEL-positive nuclei, and the superposition of blue fluorescence and green fluorescence indicated apoptotic cells (magnification, ×600; scale bar = 40 *μ*m, *n* = 15). The representative images were from three independent experiments. For each independent experiment, at least 5 fields were observed and counted under a confocal laser scanning microscope in each group. (d) Colorimetric assay was used to detect the activity of caspase-3 in H9C2 cells transfected with caspase-3-C163S-His plasmid (*n* = 11). (e) TUNEL assay was used to detect the apoptosis of H9C2 cells transfected with caspase-3-C163S-His plasmid. The blue fluorescence indicated the nuclei, the green fluorescence indicated the TUNEL-positive nuclei, and the superposition of blue fluorescence and green fluorescence indicated apoptosis cells (magnification, ×600; scale bar = 40 *μ*m, *n* = 15). The representative images were from three independent experiments. For each independent experiment, at least 5 fields were observed and counted under a confocal laser scanning microscope in each group. The data were expressed as mean ± SEMs, ^∗∗^*p* < 0.01. Ctrl: control; NaHS: sodium hydrosulfide; NaHS+DTT: sodium hydrosulfide+dithiothreitol; DOX: doxorubicin; DOX+NaHS: doxorubicin+sodium hydrosulfide.

It was reported that Cys-163 was the active catalytic site of caspase-3 [42]. Here, we investigated the exact site on which H_2_S persulfidated caspase-3, targeting Cys-163. In the experiment, its two neighboring cysteine residues were used as the control residues. The caspase-3 wild-type plasmid (caspase-3-WT-His) and three caspase-3 mutant plasmids, including caspase-3-C163S-His, caspase-3-C148S-His, and caspase-3-C170S-His plasmids, were transfected into caspase-3 knockdown H9C2 cells, respectively. Each type of cells was, respectively, divided into control, NaHS, and NaHS+DTT groups. The results indicated that the persulfidation of caspase-3 in the NaHS group was greater than that of the control H9C2 cells transfected with caspase-3-WT-His, caspase-3-C148S-His, or caspase-3-C170S-His plasmid, which was abolished by the DTT (*p* all <0.01). However, there was no difference in the ratio of persulfidated caspase-3/caspase-3 with or without NaHS treatment in the cells transfected with the caspase-3-C163S-His plasmid (Figure 4(a)). These results suggested that Cys-163 might be the target residue on which H_2_S persulfidated caspase-3.

Furthermore, to examine the significance of caspase-3 persulfidation in the H_2_S-inhibited caspase-3 activation and apoptosis of H9C2 cells treated by DOX, we compared the inhibitory effect of H_2_S on caspase-3 activity and apoptosis between H9C2 cells transfected with caspase-3-WT-His and caspase-3-C163S-His plasmids. The results revealed that in the H9C2 cells transfected with caspase-3-WT-His plasmid, H_2_S donor inhibited DOX-stimulated activation of caspase-3 and the increase in the proportion of TUNEL-positive cells (*p* < 0.01) (Figures 4(b) and 4(c)). However, in the cells transfected with the caspase-3-C163S-His plasmid, neither caspase-3 activity nor cell apoptosis was affected by the treatment of H_2_S donors (Figures 4(d) and 4(e)).

These above findings suggested that H_2_S persulfidated caspase-3 at Cys-163 to inhibit the DOX-induced caspase-3 activation and accordingly cardiomyocyte apoptosis.

## 4. Discussion

**Figure 5:**
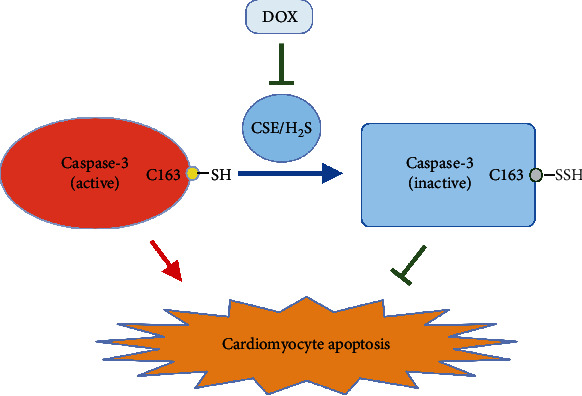
A schematic diagram showing a novel cardioprotective mechanism by which endogenous H_2_S inhibits DOX-induced cardiomyocyte apoptosis. Endogenous H_2_S inactivates caspase-3 via the persulfidation of caspase-3 at Cys-163, thereby blocking cardiomyocyte apoptosis.

In the present study, we revealed that DOX induced cardiomyocyte apoptosis by downregulating the CSE/H_2_S pathway. Furthermore, sufficient endogenous H_2_S persulfidated caspase-3 to inhibit its activity, alleviating DOX-stimulated apoptosis of cardiomyocytes. The Cys-163 site of caspase-3 acted as the target residue for H_2_S to persulfidate caspase-3 protein (Figure 5).

DOX, an efficient anthracycline antitumor agent, can cause cardiomyopathy, cardiac damage, arrhythmia, and heart failure. Cardiomyocyte apoptosis is an important pathological basis of DOX-induced cardiomyopathy [3, 6, 9]. Previous studies have shown that DOX directly damages DNA, generates reactive oxygen species through protein/DNA interactions, and leads to cell death [43]. DOX also inhibits topoisomerase II, resulting in double-strand DNA breakage and hindering cell replication and transcription [44]. However, the molecular mechanisms underlying DOX-stimulated cardiomyocyte apoptosis have not yet been entirely clear and need to be further explored.

Endogenous H_2_S is a novel gasotransmitter identified after nitric oxide (NO) and carbon monoxide (CO) [27–30, 45, 46]. It is mainly generated endogenously by CSE catalyzed in the cardiovascular system [47]. H_2_S is widely involved in the pathophysiological processes in the body, especially in the cardiovascular system. In preliminary animal experiments, H_2_S improved cardiac function in DOX-induced cardiomyopathy in rats and reduced myocardial damage. H_2_S donors have been demonstrated to significantly reduce cardiomyocyte apoptosis [36]. In this study, we explored the regulatory effect of H_2_S on the DOX-stimulated apoptosis of cardiomyocytes and its molecular mechanisms.

It has been reported that the steady-state plasma concentration of DOX is 22.6-334 ng/mL (0.03-0.5 *μ*mol/L) in patients treated with DOX [48]. Therefore, we used the CCK-8 method to assess the effect of different concentrations (0-0.5 *μ*mol/L) of DOX on the viability of H9C2 cells, and the data indicated that 0.3 *μ*mol/L DOX administration reduced H9C2 cell viability by 50%. The TUNEL assay indicated that the apoptotic rate of H9C2 cells exposed to 0.3 *μ*mol/L DOX was as high as 85.94%, thus confirming the successful establishment of the apoptosis model induced by 0.3 *μ*mol/L DOX for 24 h.

The results demonstrated that DOX induced apoptosis of H9C2 cells and significantly downregulated the endogenous H_2_S production and the expression of endogenous synthase CSE. Previous studies showed that reduced H_2_S content could cause cardiomyocyte apoptosis during ischemia/reperfusion injury [49, 50]. Treatment with H_2_S donors to restore H_2_S content in heart tissue significantly inhibited isoprenaline-induced cardiomyocyte apoptosis [51]. In addition, endogenous H_2_S was involved in the development of diabetic cardiomyopathy [52, 53]. These findings suggest that endogenous H_2_S has a strong myocardial protective effect and inhibits cardiomyocyte apoptosis in the pathogenesis of cardiac diseases. In the present study, H_2_S donor treatment and CSE overexpression were used to increase the content of H_2_S, which inhibited the apoptosis of DOX-stimulated cardiomyocytes, suggesting that endogenous H_2_S/CSE downregulation might be an important mechanism for DOX-induced cardiomyocyte apoptosis.

The caspase family is synthesized in the form of inactive proenzymes, and the activated caspase molecules play significant roles in the apoptotic pathway in cardiomyocytes [54–56], gradually disintegrating cell components through the cleavage of specific substrates that can reconstruct the cytoskeleton and degrade nuclei. Furthermore, apoptotic cells exhibit shrunken morphology, condensed chromatin, and finally internuclear chromosomal DNA fragmentation [57]. Previous studies have shown that members of the caspase family are widely involved in the process of DOX-induced apoptosis in cardiomyocytes [12, 55, 56, 58, 59]. In this study, we found that DOX activated almost all members of the caspase family in cardiomyocytes, and H_2_S donor NaHS intervention inhibited caspase-2, caspase-3, and caspase-9 activities, exerting the strongest inhibitory effect on caspase-3.

Among the caspase family members, caspase-3 is a critical regulator of apoptosis and serves as the hub of endogenous and exogenous apoptotic pathways, playing an important role at the final stage of apoptosis [60]. Therefore, caspase-3 has attracted widespread attention since its discovery in 1994 [61]. Caspase-3 precursor is a single-chain inactive peptide with a molecular weight of 32 kDa. Caspase-3 can be activated by its upstream regulators including caspase-8 or caspase-9 to produce active tetramer structures composed of two large (17 kD) and small (12 kD) subunits [62]. Activated caspase-3 further cleaves its downstream PARP into two fragments. Then, cleaved PARP fails to inhibit the activity of Ca^2+^/Mg^2+^-dependent nucleic acid endonuclease, leading to DNA fragmentation and programmed apoptosis [63]. In the present study, we found that either H_2_S donors or overexpression of CSE blocked the effects of DOX on caspase-3 activation and PARP cleavage. The results suggest that caspase-3 might be a target of H_2_S to prevent cardiomyocyte apoptosis. However, the mechanism by which H_2_S inhibits caspase-3 activity remains unclear.

Caspase-3 activity depends on a conserved QACRG pentapeptide sequence, in which a reducing sulfhydryl group at the cysteine residue is acquired to maintain the activity of caspase-3 [64]. As a strong reducing agent, DTT can block the oxidation process of sulfhydryl group and keep it in a reduced state (R-SH) [65]. In this study, we found that DTT blocked the suppressive effect of H_2_S on caspase-3 activation and apoptosis in DOX-treated cardiomyocytes, suggesting that the suppressive effect of H_2_S on the activation of caspase-3 and cell apoptosis might be related to the sulfhydryl group on the caspase-3. It was reported that H_2_S regulates target protein function by persulfidation, a posttranslational modification occurring at a specific sulfhydryl group, which converted a reduced sulfhydryl group (R-SH) to a persulfidated sulfhydryl group (R-SSH) and then affected the structure and function of the protein [34, 66, 67]. Therefore, we investigated the persulfidation of caspase-3 in NaHS-treated H9C2 cells and purified human caspase-3 protein. As expected, both the cellular and cell-free experimental findings suggested that H_2_S persulfidated caspase-3, which could be reversed by DTT treatment.

Furthermore, it is important to demonstrate the exact site at which H_2_S persulfidates caspase-3 to elucidate the cardioprotective mechanism responsible for H_2_S. Previous studies reported that Cys-163 is the active site catalyzing protease hydrolysis [62]. The glutathionylation of caspase-3 inhibits the activation of caspase-3 [26]. NO also exerts antiapoptotic effects through the nitrosylation of caspase-3 at Cys-163 [68–72]. Therefore, in this study, Cys-163 was selected as the candidate residue of H_2_S-induced persulfidation of caspase-3, while Cys-148 and Cys-170, the two neighbor cysteine residues, were used as control residues. Interestingly, we found that when Cys-163 in caspase-3 was replaced with Ser, the persulfidation of caspase by H_2_S no longer occurred. On the contrary, neither the mutation at Cys-148 nor Cys-170 impacted the persulfidation of caspase-3 by H_2_S. Moreover, H_2_S did not affect caspase-3 activity and cardiomyocyte apoptosis in DOX-treated H9C2 cells transfected with the mutated caspase-3-C163S-His plasmid. These data demonstrated that H_2_S inhibited the apoptosis of DOX-stimulated cardiomyocytes by persulfidating caspase-3 at Cys-163 to suppress caspase-3 activation.

Regarding the mechanisms by which H_2_S-induced persulfidation of caspase-3 at Cys-163 inhibited the caspase-3 activity, we speculated the following possibilities. (1) The cleavage of substrate peptide bonds catalyzed by the caspase-3 depends on the attack of the nucleophile thiol group of catalytic Cys-163 on the aspartic acid at P1 position [73]. Based on the above characteristics of activated caspase-3, caspase-3 inhibitors were developed to inhibit caspase-3 activity by forming a covalent bond with the thiol group of Cys-163 [74]. Therefore, we speculated that the persulfidated sulfhydryl group of Cys-163 induced by H_2_S might change the nucleophilic ability of Cys-163 and then inhibit the caspase-3 activity. (2) Acting as an effector caspase, caspase-3 cannot be autoactivated. However, one activated caspase-3 could cleave another procaspase-3 to form an active caspase-3 [75] and then promote caspase-3 activation cascade. Therefore, the block of procaspase-3 cleavage might also be involved in the mechanism responsible for the inhibitory effect of caspase-3 persulfidation on the caspase-3 activity. In addition, the detection of caspase-3 persulfidation and activity in the heart from DOX-treated animals might also strengthen the causality relationship between the persulfidation of caspase-3 and the inhibition of caspase-3 activity, which merits further investigation.

## 5. Conclusion and Perspective

In summary, the present study revealed a novel mechanism by which H_2_S inhibited DOX-induced apoptosis of cardiomyocytes. We first showed that H_2_S inhibited the activity of caspase-3 by persulfidating Cys-163 residue on caspase-3, suppressing apoptosis in DOX-treated cardiomyocytes. Therefore, the results of this study clearly revealed the protective effect of endogenous H_2_S on the cardiotoxicity induced by DOX and its mechanisms, providing a new idea and target for the potential therapeutic intervention against DOX-stimulated cardiomyopathy.

The effect of H_2_S on the antineoplastic efficacy of DOX should be paid great attention when it is considered as a potential treatment strategy in the anticancer therapy. Chegaev et al. developed new DOX derivatives and designed a series of H_2_S-donating DOXs which continuously released H_2_S in DMEM culture and human serum. Furthermore, H_2_S-donating DOXs exhibited a stronger cytotoxicity than DOX alone in both DOX-sensitive and DOX-resistant osteosarcoma cells via impairing membrane P glycoprotein transporter to block DOX efflux and enhance DOX effectiveness [76]. Wang et al. found that compared with DOX only, the implantation of DOX-ZnS@SiO_2_ fibrous mesh significantly shrank the tumor size and made a more seriously histological damage in the Huh7 mouse tumor model by releasing H_2_S to enhance the proapoptotic effect of DOX [77]. The abovementioned findings suggested that cotreatment with H_2_S effectively enhanced chemotherapeutic efficacy of DOX *in vitro* and *in vivo*, which further supported the potential clinical application of H_2_S in the antineoplastic treatment.

Another important issue that should be concerned is about the PARP. PARP, acting as a DNA damage sensor and signal transducer, plays a key role in the DNA repair, and therefore, PARP inhibitor has been developed to block the DNA repair in the cancer cells treated with chemotherapy or radiotherapy [78]. Pettitt et al. found that siRNA-mediated PAPR1 deficiency abolished the cytotoxicity of PARP1 inhibitors in both human CAL51 cells and DLD1 cells, suggesting that the normal expression of the intact PARP1 is essential for anticancer efficacy of PARP1 inhibitor [79]. In our present study, the integrity of PARP1 was preserved by H_2_S treatment since H_2_S suppressed the activated caspase-3-mediated cleavage of PARP1. Therefore, we speculated that H_2_S might cooperate with PARP inhibitor to enhance its anticancer effect in the cancer cells although the above speculation needs to be confirmed in the future.

## Data Availability

The data used to support the findings of the study are available from the corresponding authors upon request.
